# MH84 improves mitochondrial dysfunction in a mouse model of early Alzheimer’s disease

**DOI:** 10.1186/s13195-018-0342-6

**Published:** 2018-02-13

**Authors:** Maximilian Pohland, Maren Pellowska, Heike Asseburg, Stephanie Hagl, Martina Reutzel, Aljoscha Joppe, Dirk Berressem, Schamim H. Eckert, Mario Wurglics, Manfred Schubert‐Zsilavecz, Gunter P. Eckert

**Affiliations:** 1 Institute of Pharmacology, Goethe University, Frankfurt, Germany; 2 Institute of Pharmaceutical Chemistry, Goethe University, Frankfurt, Germany; 3 Institute of Nutritional Sciences, Justus-Liebig-University, Giessen, Germany

**Keywords:** Alzheimer’s disease, Mitochondrial dysfunction, PPAR gamma activator, PGC-1 alpha, APP processing, Amyloid-beta

## Abstract

**Background:**

Current approved drugs for Alzheimer’s disease (AD) only attenuate symptoms, but do not cure the disease. The pirinixic acid derivate MH84 has been characterized as a dual gamma-secretase/proliferator activated receptor gamma (PPARγ) modulator in vitro. Pharmacokinetic studies in mice showed that MH84 is bioavailable after oral administration and reaches the brain. We recently demonstrated that MH84 improved mitochondrial dysfunction in a cellular model of AD. In the present study, we extended the pharmacological characterization of MH84 to 3-month-old Thy-1 AβPP_SL_ mice (harboring the Swedish and London mutation in human amyloid precursor protein (APP)) which are characterized by enhanced AβPP processing and cerebral mitochondrial dysfunction, representing a mouse model of early AD.

**Methods:**

Three-month-old Thy-1 AβPP_SL_ mice received 12 mg/kg b.w. MH84 by oral gavage once a day for 21 days. Mitochondrial respiration was analyzed in isolated brain mitochondria, and mitochondrial membrane potential and ATP levels were determined in dissociated brain cells. Citrate synthase (CS) activity was determined in brain tissues and MitoTracker Green fluorescence was measured in HEK293-AβPPwt and HEK293-AβPPsw cells. Soluble Aβ_1–40_ and Aβ_1–42_ levels were determined using ELISA. Western blot analysis and qRT-PCR were used to measure protein and mRNA levels, respectively.

**Results:**

MH84 reduced cerebral levels of the β-secretase-related C99 peptide and of Aβ40 levels. Mitochondrial dysfunction was ameliorated by restoring complex IV (cytochrome-c oxidase) respiration, mitochondrial membrane potential, and levels of ATP. Induction of PPARγ coactivator-1α (PGC-1α) mRNA and protein expression was identified as a possible mode of action that leads to increased mitochondrial mass as indicated by enhanced CS activity, OXPHOS levels, and MitoTracker Green fluorescence.

**Conclusions:**

MH84 modulates β-secretase processing of APP and improves mitochondrial dysfunction by a PGC-1α-dependent mechanism. Thus, MH84 seems to be a new promising therapeutic agent with approved in-vivo activity for the treatment of AD.

**Electronic supplementary material:**

The online version of this article (10.1186/s13195-018-0342-6) contains supplementary material, which is available to authorized users.

## Background

Alzheimer’s disease (AD) is an age-related neurodegenerative disease and the primary cause for dementia in the elderly which represents a growing public health issue [1]. After a long prodromal phase, AD manifests itself clinically by a progressive cognitive decline followed by gradual personality changes [2]. Currently, there is no proven disease-modifying treatment available [3]. Interventions with current approved drugs, if started early enough, may at best temporarily slow down the progression but cannot impede dementia [4]. Thus, new therapeutic strategies are in the focus of drug discovery programs [5, 6]. Neuropathological hallmarks of AD are extracellular amyloid plaques and intracellular neurofibrillary tangles [7, 8]. Amyloid plaques are composed of beta-amyloid (Aβ) protein, processed from the beta-amyloid precursor protein (AβPP) by initial β-secretase cleavage resulting in peptide C99. Subsequent cleavage of C99 by the γ-secretase complex results in the formation of Aβ species of different lengths ranking from Aβ39 to Aβ43 [9]. β-Secretase, γ-secretase, and Aβ itself were identified as pharmacological targets [10]. Peroxisome proliferator-activated receptor gamma (PPARγ) represents another target for AD treatment [11–13]. A small pilot study that tested PPARγ agonist pioglitazone in patients with mild AD accompanied with type II diabetes exhibited cognitive and functional improvements [14]. Another preliminary study with rosiglitazone also showed preserved cognition in patients with early AD [15]. However, a large randomized clinical trial failed to show clinical efficacy of rosiglitazone in AD. In this trial, also no treatment difference in cognitive testing was detected for the competitor donepezil [16].

Beside its effects on AβPP processing and Aβ-mediated cell death, including reduced BACE1 expression and transcription, decreased BACE1 promoter activity, and a clearance mechanism for Aβ [17–19], PPARγ agonists provide neuroprotection by improving mitochondrial dysfunction in models of neurodegenerative diseases [20, 21]. As an early event in AD pathogenesis [22, 23] mitochondrial dysfunction contributes to an impairment of the energy metabolism, defects in key respiratory enzyme activity/function, accumulation/generation of mitochondrial reactive oxidative species (ROS), induction of apoptosis, and altered mitochondrial biogenesis and dynamics [24, 25]. These observations led to the hypothesis that impaired mitochondrial function, associated with reduced energy metabolism and enhanced oxidative stress as well as synaptic dysfunction, represents a common final pathway of all specific (genetic) and nonspecific risk factors for the development of AD [22, 26, 27]. This concept was put forward in the “mitochondrial cascade hypothesis” first proposed more than 10 years ago by Swerdlow et al. [28, 29]. This concept suggests that mitochondria at least mediate or possibly even initiate pathologic molecular cascades in AD [30].

Peroxisome proliferator-activated receptor-gamma coactivator alpha (PGC-1α) is a nuclear factor that regulates mitochondrial biogenesis in response to diverse environmental stimuli [31]. PGC-1α has been associated with AD [32] and PGC-1α messenger RNA expression was reported to be significantly decreased as a function of progression of clinical dementia in the AD brain [33]. It has been reported that PGC-1α could protect cells against oxidative stress and reduce mitochondrial dysfunction [32].

**Fig. 1:**
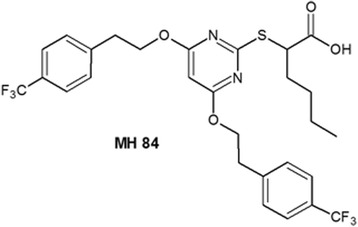
Molecular structure of MH84 (ethyl 2-(4,6-bis(4-(trifluoromethyl)phenethoxy)pyrimidin-2-yl-thio)hexanoate) [12]

The pirinixic adic derivate MH84 (ethyl 2-(4,6-bis(4-(trifluoromethyl)-phenethoxy)pyrimidin-2-yl-thio)hexanoate) (Fig. 1) was developed from the optimization of a novel structural class of dual γ-secretase/PPARγ modulators [12, 34]. Pharmacokinetic properties of MH84 were investigated after oral administration of a single dose of 12 mg/kg b.w. in C57-Bl/6 mice and published previously (Table 1) [35]. In brain tissue a constant level of 300 to a maximum 320.64 ng/g was found after 1.5–6 hours [35]. We recently demonstrated that MH84 improved mitochondrial dysfunction in a cellular model of AD [36]. In the present study, we extended the pharmacological characterization of MH84 to 3-month-old Thy-1 AβPP_SL_ mice which are characterized by enhanced AβPP processing and cerebral mitochondrial dysfunction, representing a mouse model of early AD [37, 38].

**Table 1:** Pharmacokinetic parameters of MH84

Parameter	Value
*C*_max_ (μg/g plasma)	10.90
*T*_max_ (h)	3.00
AUC_48h_ (μg h/ml)	89.58
*T*_1/2_ (h)	3.24
CL (ml/min/kg)	2.23
Vd (L/kg)	0.63

Plasma levels of MH84 were determined using HPCL after single oral gavage of 12 mg/kg MH84. The following pharmacokinetic parameters were adopted from our previous publication (for details, please refer to Pellowska et al. [35]): *C*_max_, maximum concentration; *T*_max_, time to reach *C*_max_; AUC_48h_, area under the curve until 48 h; *T*_1/2_, half-life time; CL, clearance; Vd, volume of distribution

*MH84* ethyl 2-(4,6-bis(4-(trifluoromethyl)phenethoxy)pyrimidin-2-yl-thio)hexanoate

## Methods

### Chemicals

Unless otherwise stated, chemicals were obtained from Sigma-Aldrich (Taufkirchen, Germany). MH84 (ethyl 2-(4,6-bis(4-(trifluoromethyl)phenethoxy)pyrimidin-2-yl-thio)hexanoate) was synthesized according to literature procedures [12]. Analytical NMR data of the batch used for the current study are as follows (see also Additional file 1: Figure S1):

^1^H-NMR (300.13 MHz, (CD3)2SO): δ = 0.78–0.83 (t, 3H, *J* = 7.1 Hz, Bu-CH3), 1.22–1.37 (m, 4H, Bu-CH2), 1.74–1.92 (m, 2H, Bu-CH2), 3.06–3.11 (t, 4H, *J* = 6.6 Hz, Ph-CH2), 4.27–4.30 (t, 1H, *J* = 7.1 Hz, S-CH), 4.48–4.53 (t, 4H, *J* = 6.6 Hz, Pyr-O-CH2), 5.87 (s, 1H, Pyr-CH), 7.49–7.52 (d, 4H, *J* = 8.0 Hz, Ph-C2/6), 7.63–7.66 (d, 4H, *J* = 8.0 Hz, Ph-C3/5), 12.82 (s/br, 1H, COOH). ^13^C-NMR (75.44 MHz, (CD3)2SO): δ = 13.64 (Bu-CH3), 21.67 (Bu-CH2), 28.92 (Bu-CH2), 30.91 (Bu-CH2), 34.16 (2C, Ph-CH2), 47.46 (S-CH), 66.54 (2C, Pyr-O-CH2), 85.97 (Pyr-C5), 124.99 (4C, Ph-C3/5), 125.14–127.74 (2C, -CF3), 129.69 (4C, Ph-C2/6), 143.06 (2C, Ph-C1), 168.95 (Pyr-C2), 169.97 (2C, Pyr-C4/6), 172.23 (COOH). MS (ESI): *m/e* = 601.4 (M–1). Anal. (C_28_H_28_F_6_N_2_O_4_S (602.17)) C, H, N, S: ber, C 55.81, H 4.68, N 4.65, S 5.32; gef, C 56.10, H 4.86, N 4.40, S 5.40; Abw, C 0.29, H 0.18, N 0.25, S 0.08.

### Animals and treatment

Twenty-two C57BL/6 mice at the age of 3 months bearing the human Swedish (S: KM595/596NL) and London (L: V717I) mutations in the 751 amino acid form of human amyloid-beta precursor protein (AβPP) under control of a murine Thy-1 promoter were randomly divided into two groups of 11 Thy-1 AβPP_SL_ mice (Thy-1 AβPP_SL (control)_ and Thy-1 AβPP_SL (MH84)_) [39]. A number of 11 wild-type littermate animals were used as nontransgenic controls. Each group consisted of six females and five males. All animals were genotyped by tail biopsies and polymerase chain reaction before and after the experiments (data not shown). Animals were housed according to the German guidelines for animal care with access to water and food ad libitum. They were maintained on a 12-h light/dark cycle. Thy-1 AβPP_SL (MH84)_ mice received 12 mg/kg b.w. MH84 by oral gavage once a day for 21 days. MH84 was diluted in polyethylene glycol 400 (PEG400). Control groups received an equal amount of PEG400. Health was assessed daily and PEG400 had no adverse effects. The mice were sacrificed by decapitation after cervical dislocation and the brains were quickly dissected on ice after removal of the cerebellum, the brain stem, and the olfactory bulb. All experiments were carried out by individuals with appropriate training and experience according to the requirements of the Federation of European Laboratory Animal Science Associations and the European Communities Council Directive (Directive 2010/63/EU). Experiments were approved by the regional authority (Regierungspraesidum Darmstadt; #V54-19 c 20/15-FU_K_5003).

### Preparation of isolated brain mitochondria

Brain mitochondria were isolated from one hemisphere as reported previously [40]. Briefly, the cerebrum was homogenized using a potter equipped with a Teflon^®^ pistil in 2 ml mitochondrial respiration medium (MiR05) containing a protease inhibitor cocktail (PI, complete; Roche, Mannheim, Germany). The resulting homogenate was centrifuged to remove the nuclei and other cell debris (1400 × *g*, 7 min, 4 °C). The low-speed centrifugation step was repeated once (for 3 min) with the supernatant followed by a high-speed centrifugation step (10.000 × *g*, 5 min, 4 °C) to collect a pellet enriched in mitochondria. This pellet was resuspended in 1 ml ice-cold MiR05 + PI and centrifuged (1400 × *g*, 3 min, 4 °C). The supernatant was centrifuged once again at high speed (10.000 × *g*, 5 min, 4 °C) to obtain the mitochondrial fraction. The resulting pellet was then dissolved in 250 μl MiR05 + PI. The obtained solution of isolated mitochondria was then used for high-resolution respirometry (80 μl). The remaining solution was immediately frozen in liquid nitrogen for citrate synthase (CS) activity (120 μl) and protein determinations (50 μl) and stored at –80 °C.

### High-resolution respirometry

Mitochondrial respiration was analyzed as reported previously [40]. Briefly, 80 μl of isolated mitochondria in MiR05 were injected into a chamber of the Oxygraph-2 k at 37 °C (Oroboros, Innsbruck, Austria), and a complex protocol (developed by Erich Gnaiger, Innsbruck, Austria) containing different substrates, uncouplers, and inhibitors was carried out [41]. The capacity of oxidative phosphorylation (OXPHOS) was determined with complex I-related substrates (CI_OXPHOS_) pyruvate (5 mM), malate (2 mM), and adenosine 5′-diphosphate (ADP) (2 mM) followed by addition of succinate (10 mM, CI + II_OXPHOS_). Mitochondrial integrity was examined via addition of cytochrome c (10 μM). Subsequently, oligomycin (2 μg/ml) was added to reveal leak respiration (leak omy). The maximum capacity of the electron transfer system (ETS) was achieved by titration of carbonyl cyanide *p*-(trifluoromethoxy) phenylhydrazone (FCCP). Complex II respiration in the noncoupled state (CII_ETS_) was measured after adding rotenone (0.5 μM) into the chambers. Residual oxygen consumption (ROX) was determined after inhibition of complex III by addition of antimycin A (2.5 μM) and was subtracted from all respiratory parameters. Cyclooxygenase activity (CIV_ETS_) was measured after by applying 0.5 mM tetramethylphenylenediamine (TMPD) as an artificial substrate of CIV and 2 mM ascorbate to keep TMPD in the reduced state. The autoxidation rate was determined after the addition of sodium azide (≥100 mM). For data analysis, DatLab 5.1.1.7 software (Oroboros) was used. Data of mitochondrial respiration were normalized to CS activity.

### Preparation of dissociated brain cells for in-vitro studies

Dissociated brain cells (DBC) were isolated from one hemisphere as reported previously [37, 40]. Briefly, after preparation, DBC were resuspended in 4.5 ml DMEM without supplements. The suspension was seeded in 250-μl aliquots into a 24-well plate with 12 replicates (for measurement of mitochondrial membrane potential) or in 50-μl aliquots into a 96-well plate with 12 replicates (for measurement of ATP level). Afterward the cells were maintained for 3 h at 37 °C in a humidified incubator, gassed with 5% CO_2_. A 1-ml aliquot of the remaining cell suspension was collected and immediately frozen at –80 °C for protein determination.

### Determination of mitochondrial membrane potential

The potential of the inner mitochondrial membrane (MMP) was determined in DBC as described previously using the cell-permeable fluorescent dye rhodamine 123 (R123) with a VICTOR™ X3 2020 Multilabel Counter (Perkin Elmer, Rodgau, Germany) [40].

### Determination of ATP levels

ATP levels were determined in DBC using the ViaLight^®^ Plus bioluminescence assay according to the manufacturer’s instructions (Lonza, Walkersville, USA) with a VICTOR™ X3 Multilabel Counter (Perkin Elmer), as described previously [37, 40].

### Citrate synthase activity

CS activity was determined in isolated mitochondria as described previously [40]. A frozen subsample of the isolated mitochondria dissolved in MiR05 was thawed on ice. A reaction medium containing 100 μl of 0.1 mM 5,5′-dithiobis-(2-nitrobenzoic acid) (DTNB), 25 μl of 10% Triton X-100, 50 μl of 10 mM oxaloacetate, 25 μl of 12.2 mM acetyl coenzyme A, and 790 μl purified water was mixed and preheated at 30 °C for 5 min. Subsequently, 10 μl of the isolated mitochondria was added to the reaction medium and transferred into a 10-mm quartz cuvette (Hellma^®^ Analytics, Muellheim, Germany). CS activity was assessed at 412 nm using a GENESYS 5 spectrophotometer (Spectronic via Thermo Fisher Scientific, Waltham, MA, USA) and normalized to protein content. Measurements were performed in duplicate.

### Cell culture

Human embryonic kidney (HEK) 293 cells containing human wild-type AβPP (HEK293_AβPPwt_) and HEK293_AβPPsw_ cells transfected with DNA constructs harboring human mutant AβPP (AβPPsw, KM670/671NL) gene were cultured in Dulbecco’s modified Eagle’s medium (DMEM) supplemented with 10% heat-inactivated fetal calf serum, 50 units/ml penicillin, 50 μg/ml streptomycin, and 400 μg/ml Geniticin (G418). Both cell lines were maintained in a humified incubator, gassed with 5% CO_2_ at 37 °C as described previously [36]. The HEK293_AβPPwt_ cell line was a kind gift received from Prof. Christian Haass, Munich, Germany.

### MitoTracker Green

MitoTracker Green (MTG) measurements were performed as described previously [42]. Briefly, 2 days before the measurement 100,000 cells/well of HEK293_AβPPwt_ cells and HEK293_AβPPsw_ cells were seeded into a 24-well plate. On the next day cell medium was incubated for 24 h with 0.1 μM MH84. For solvent control DMSO (0.05%) was used. Afterward the cells were washed with 500 μl Hank’s Balanced Salt Solution buffer (HBSS) (pH 7.4, 37 °C supplemented with Mg^2+^,Ca^2+^ and HEPES) and spiked with 1 μM MTG (Invitrogen, Camarillo, USA) and incubated for 60 min. After adding fresh HBSS buffer to the cells, fluorescence was measured at an excitation wavelength of 490 nm and an emission wavelength of 516 nm in a VICTOR™ X3 Multilabel Counter (Perkin Elmer). When comparing HEK293_AβPPwt_ cells and HEK293_AβPPsw_ cells, fluorescence values were normalized to protein content.

### Determination of soluble Aβ

The content of soluble Aβ_1–40_ and Aβ_1–42_ in supernatants was detected by specific solid phase sandwich enzyme-linked immunosorbent assays (ELISA; Life Technologies, Carlsbad, CA, USA) following the instructions of the supplier. Brain material was homogenized in 10 times the amount of phosphate-buffered saline containing a protease inhibitor cocktail. After homogenization, samples were centrifuged (15,000 × *g*, 30 min, 4 °C) and the supernatants were transferred to a fresh reaction vessel and stored at –80 °C prior to analysis. Total protein content was determined using a Pierce™ BCA Protein Assay Kit (Thermo Scientific, Rockford, IL, USA).

### Western blot analysis

Western blot analysis was carried out in brain tissue homogenates as reported previously [36]. Briefly, brain samples were homogenized in lysis buffer. Total protein content was determined using a Pierce™ BCA Protein Assay Kit (Thermo Scientific). Band analysis was performed using ChemiDoc XRS system (BioRad, Munich, Germany). The following antibodies were used: full-length AβPP (fl-AβPP) and C-terminal fragments (CTFs) were detected using AβPP C-Terminal Fragment (C1/6.1) antibody (SIG-39152; Covance, Princeton, NJ, USA). Detection of respiratory system complexes was carried out by MitoProfile^®^ Total OXPHOS Rodent WB Antibody Cocktail (ab110413; abcam, Cambridge, UK). As primary antibody for synaptic markers, Anti-BDNF (ab72439; abcam), Anti-GAP43 (ab12274; abcam), and Anti-synaptophysin (ab32127; abcam) were used. To detect protein expression of PGC-1α in the brain, Anti-PGC-1α antibody (ab106814; abcam) was used. Anti-tubulin antibody (ab6160; abcam) and Anti-glyceraldehyde 3-phosphate dehydrogenase (GAPDH) antibody (MAB374; Merck Millipore, Darmstadt, Germany) were used to verify equal protein loading.

### Quantitative real-time PCR

qRT-PCR measurements were carried out as described previously [42]. Briefly, total RNA was isolated from frozen brain tissues using the RNeasy Mini Kit (Qiagen, Hilden, Germany) according to the manufacturer’s instructions. Maximum yield of RNA was obtained using 20 mg/ml Proteinase K (Carl Roth, Karlsruhe, Germany). RNA was quantified by measuring the absorbance at 260 nm using a NanoDrop™ 2000c spectrophotometer (Thermo Fisher Scientific). Purity of RNA was assessed by the ratio of absorbance at 260 nm and 280 nm. First-strand cDNA was synthesized from 50 ng total RNA using the iScript cDNA synthesis kit (BioRad) according to the manufacturer’s instructions. Real-time PCR was performed using SYBR Green technology on a CFX96 Touch™ Real-Time PCR Detection System (BioRad). Target-specific primer pair reference genes PGK1 and B2M were purchased from biomol (Hamburg, Germany). Beta-site AβPP cleaving enzyme (BACE1) primer pairs were purchased from Bio-Rad Laboratories. PGC-1α primer pairs were designed by biomers.net (Ulm, Germany) (Table 2).

**Table 2:** Oligonucleotide primer sequences

Primer	Sequence	Accession number	Product size (bp)
B2M	5′-GGC CTG TAT GCT ATC CAG AA-3′ 5′-GAA AGA CCA GTC CTT GCT GA-3′	NM_009735.2	198
PGK1	5′-GCA GAT TGT TTG GAA TGG TC-3′ 5′-TGC TCA CAT GGC TGA CTTT TA-3′	NM_008828.2	185
BACE1	Confidential	NC_000075.6	65
PGC1α	5′-TGT CAC CAC CGA AAT CCT-3′ 5′-CCT GGG GAC CTT GAT CTT-3′	NM_008904.2	124

Prior optimization was conducted for each set of primers, which consisted of determining optimal primer concentration and template concentration and verifying the efficiency of the amplification. To confirm the specificity of the amplification, the PCR product was subjected to melting curve analysis and agarose gel electrophoresis. PCR amplification was performed in duplicate in a total reaction volume of 10 μl. The reaction mix consisted of 2 μl template, 5 μl iTaq™ Universal SYBR^®^ Green Supermix, and forward and reverse primers at the following concentrations: 50 nM (PGC-1α), 200 nM (BACE1), or 400 nM (PGK1, B2M). After a 3-min activation of Taq polymerase, amplification was allowed to proceed for 46 cycles. Each cycle consisted of denaturation at 95 °C for 10 s, annealing at 58 °C for 45 s, and extension at 72 °C for 29 s. BACE1 mRNA expression was performed in a two-step PCR amplification. After a 2-min activation of Taq polymerase, amplification was allowed to proceed for 40 cycles. Each cycle consisted of denaturation at 95 °C for 5 s and annealing and extension at 60 °C for 30 s. The one-cycle melting curve consisted of heating from 65 to 95 °C by 0.5 °C steps, with 5-s hold per step. Results were normalized to PGK1 and B2M as reference genes which are stably expressed in mouse brain [43]. Results were analyzed using the 2^–ΔΔCq^ method with target-specific amplification efficiency.

### Statistical analysis

All data are presented as means ± SEM. For statistical analysis GraphPad Prism 5.03 software (GraphPad Software, Inc., La Jolla, CA, USA) was used. For the examination of statistical significant differences between two groups a two-sided, unpaired Student’s *t* test and for multiple comparisons a one-way ANOVA with Tukey’s multiple comparison post test or a two-way ANOVA followed by Bonferroni post test was performed. *p* < 0.05, *p* < 0.01, and *p* < 0.001 were considered statistically significant.

## Results

### MH84 decreased β-secretase cleavage and Aβ40 levels

**Fig. 2:**
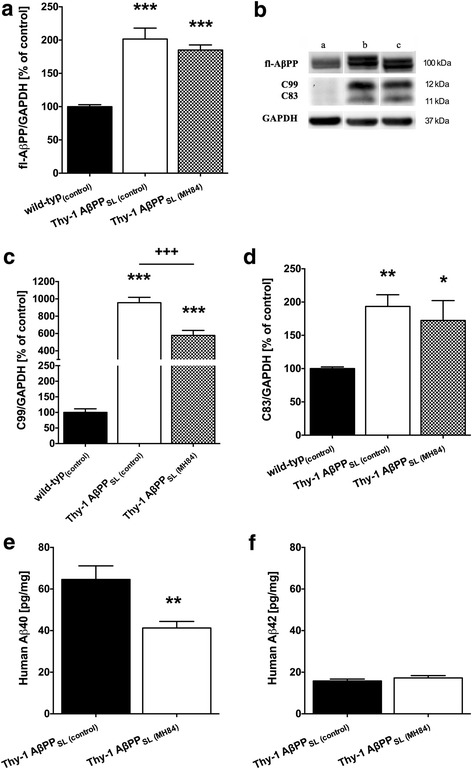
Brain levels of full-length amyloid precursor protein (**a**, **b** fl-AβPP) and its α-secretase and β-secretase cleavage products C83 and C99, respectively (**b**, **c**, **d**) detected using western blot techniques. Data represent means ± SEM from eight experiments; one-way ANOVA with Tukey’s multiple comparison post test (*p < 0.05, **p < 0.01, ***p < 0.001 against wild-type_(control)_; ^+++^*p* < 0.001 against Thy-1 AβPP_SL (control)_). Levels of soluble human β-amyloid peptides (**e** Aβ40 and **f** Aβ42) were determined using ELISA. Data represent means ± SEM. *N* = 11 (six females, five males); Student’s unpaired *t* test (**p < 0.01 against Thy-1 AβPP_SL (control)_). AβPP beta-amyloid precursor protein, Aβ amyloid-beta

Protein levels of full-length AβPP (fl-AβPP) were increased in brains of Thy-1 AβPP_SL_ mice (Fig. 2a, b). Elevated protein levels of the β-secretase cleavage products C99 and C83 were also detected (Fig. 2b–d). Approximately 68 and 18 pg/mg of human Aβ40 and Aβ42 were determined in brain tissues of Thy-1 AβPP_SL_ mice, respectively (Fig. 2e, f). Levels of soluble human Aβ40 or Aβ42 were not detectable in wild-type control mice (data not shown). Treatment with MH84 significantly decreased the β-secretase cleavage product C99 and levels of soluble human Aβ40 in brain homogenates of Thy-1 AβPP_SL_ mice (Fig. 2c, e). Expression of the β-site of APP cleaving enzyme (BACE1) significantly increased in Thy-1 AβPP_SL_ mice and administration of MH84 numerically but not significantly reduced BCAE1 mRNA levels (Additional file 2: Figure S2).

### MH84 enhanced PGC-1α expression, mitochondrial mass, and BDNF levels

**Fig. 3:**
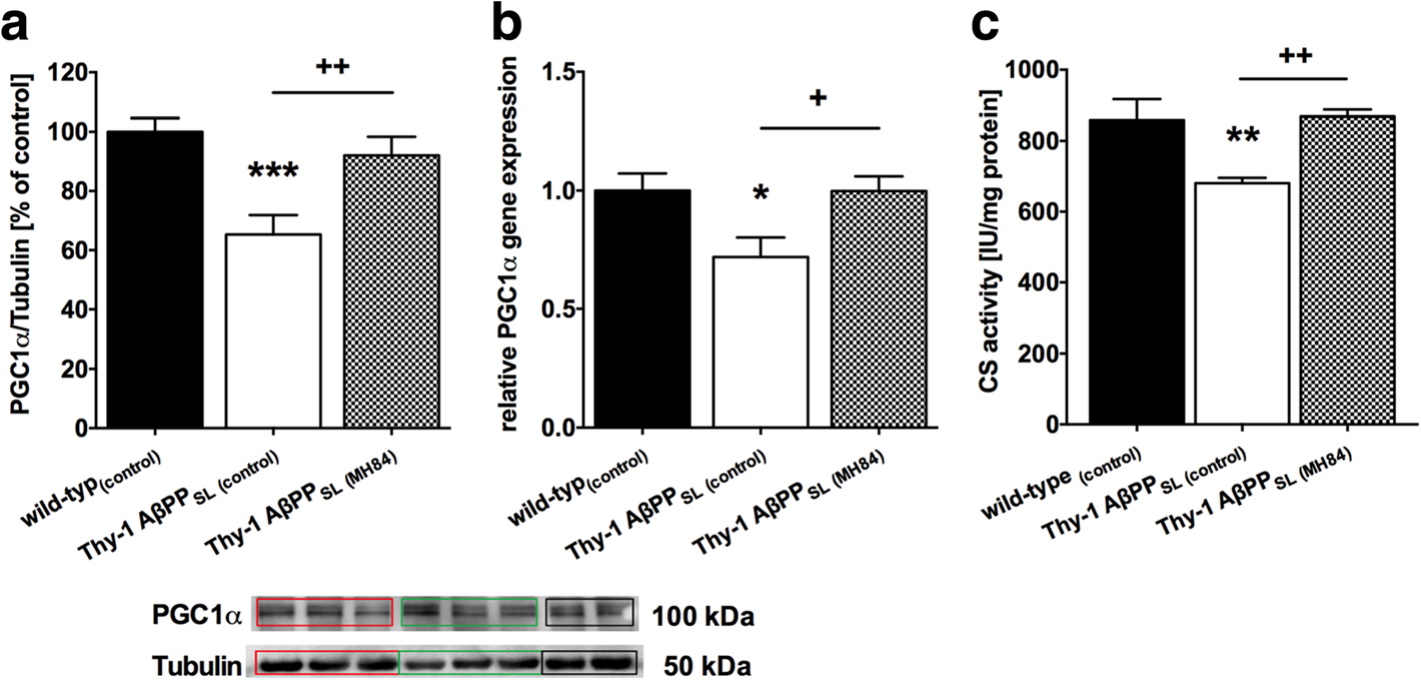
Brain levels of PGC-1α protein (**a**) and mRNA (**b**). Representative western blot assays are included in lower part of **a**. Band of PGC-1α was located in second place at 100 kDa. Tubulin used as loading control. Expression levels of mRNA were normalized to PGK1 and B2M mRNA expression. Citrate synthase (CS) activity as a marker of mitochondrial content was determined in isolated brain mitochondria using a photometrical assay (**c**). Animals belonged to three different study groups (wild-type_(control)_, Thy-1 AβPP_SL (control)_, and treatment group Thy-1 AβPP_SL (MH84)_). Data represent means ± SEM. *N* = 11 (six females, five males); one-way ANOVA with Tukey’s multiple comparison post test (*p < 0.05, ***p < 0.001 against wild-type_(control)_; ^++^*p* < 0.01, ^+^*p* < 0.05 against Thy-1 AβPP_SL (control)_). PGC-1α peroxisome proliferator-activated receptor-γ coactivator alpha, AβPP beta-amyloid precursor protein

**Fig. 4:**
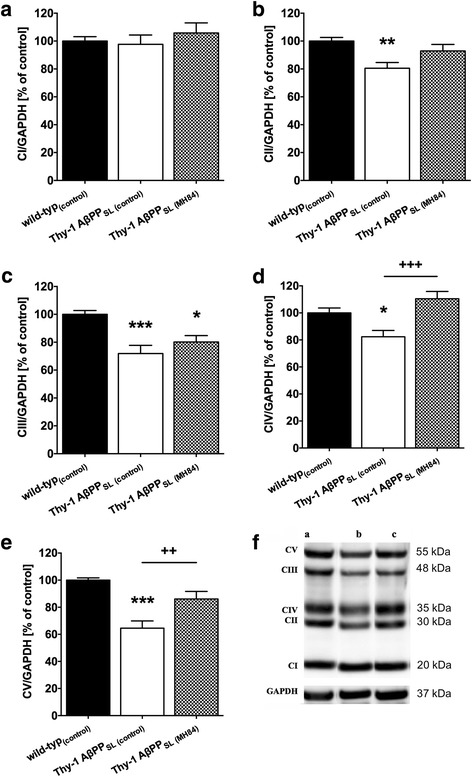
Western blot analysis of mitochondrial respiration chain complexes (**a** CI, **b** CII, **c** CIII, **d** CIV, **e** CV) in brain homogenate from wild-type_(control)_, Thy-1 AβPP_SL (control)_, and MH84-treated Thy-1 AβPP_SL_ mice. Representative western blots included in lower part of the figure (**f**). GAPDH was used as loading control. Data represent means ± SEM. *N* = 11 (six females, five males); one-way ANOVA with Tukey’s multiple comparison post test (***p < 0.001, **p < 0.01, *p < 0.05 against wild-type_(control)_; ^+++^*p* < 0.001, ^++^*p* < 0.01 against Thy-1 AβPP_SL (control)_). CI complex I (NADH reductase), CII complex II (succinate dehydrogenase), CIII complex III (cytochrome-c reductase), CIV complex IV (cytochrome-c oxidase), CV complex V (F_1_/F_0_-ATPase), AβPP beta-amyloid precursor protein

**Fig. 5:**
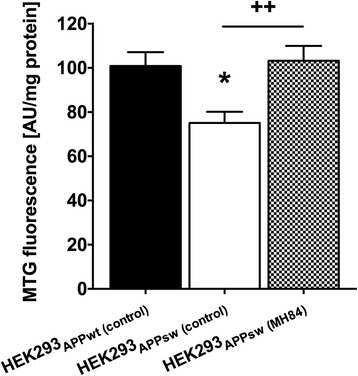
MitoTracker Green (MTG) fluorescence as a marker for mitochondrial content measured in HEK293_AβPPwt_ and HEK293_AβPPsw_ cells. Data represent means ± SEM from 12 independent experiments; one-way ANOVA with Tukey’s multiple comparison post test (*p < 0.05 against HEK293_AβPPwt (control)_; ^++^*p* < 0.01 against HEK293_AβPPsw (control)_). APP amyloid precursor protein

Protein and mRNA levels of PGC-1α were significantly decreased in the brain of Thy-1 AβPP_SL_ mice (Fig. 3a, b). Since PGC-1α represents one of the most important nuclear factors for the induction of mitogenesis [34], this finding might indicate a reduced mitochondrial mass in brains of Thy-1 AβPP_SL_ mice. Accordingly, CS activity representing a robust mitochondrial mass marker [44] was also significantly decreased (Fig. 3c). Decreased protein levels of mitochondrial respiration complexes (CII–CV) further indicate a reduced mitochondrial mass in brains of Thy-1 AβPP_SL_ mice (Fig. 4a–f). MH84 reversed protein and mRNA levels of PGC-1α as well as of CS activity (Fig. 3a–c). Moreover, MH84 significantly enhanced protein levels of CIV and CV (Fig. 4d, e). Moreover, MH84 reversed the fluorescence of the mitochondrial mass marker MitoTracker Green (MTG) in HEK293-AβPPsw cells, which further indicated that MH84 enhanced mitochondrial content (Fig. 5). HEK293-AβPPsw cells represent a cellular AD model characterized by elevated Aβ levels and mitochondrial dysfunction [36, 45, 46].

A recent report related reduced PGC-1α levels to reduced expression of brain-derived neurotrophic factor (BDNF) [47]. Western blot analysis show significantly decreased brain levels of BDNF (Additional file 2: Figure S2). The synaptic marker protein growth associated protein 43 (GAP43) was also significantly reduced in brain samples of Thy-1 APP_SL_ mice, but not of synaptophysin (Additional file 2: Figure S2). MH84 administration increased BDNF protein levels but had no effects on the synaptic marker proteins GAP43 and synaptophysin (Additional file 3: Figure S3).

### MH84 reversed mitochondrial dysfunction

**Fig. 6:**
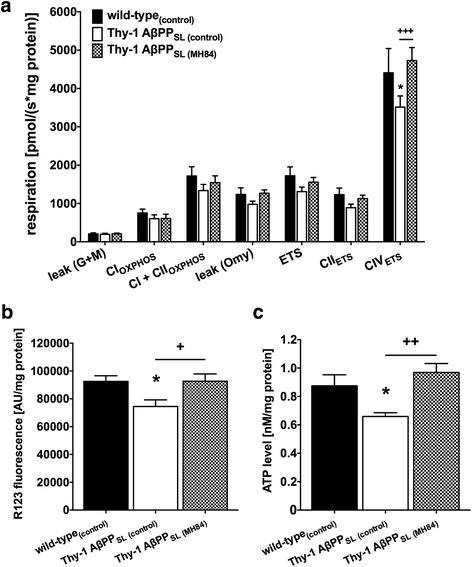
Analysis of mitochondrial respiration of isolated brain mitochondria (**a**) performed using an Oxygraph-2 k system. Animals belonged to three different study groups (wild-type_(control)_, Thy-1 AβPP_SL (control)_, and treatment group Thy-1 AβPP_SL (MH84)_). To analyze mitochondrial function different substrates, uncouplers and inhibitors were added; for details please refer to Methods. Data represent means ± SEM from 10 experiments; two-way ANOVA with Bonferroni post test (*p < 0.05 against wild-type_(control)_; ^+++^*p* < 0.001 against Thy-1 AβPP_SL (control)_). Basal mitochondrial membrane potential (MMP; R123 fluorescence) (**b**) and ATP levels (**c**) of mitochondria measured in dissociated brain cells. MMP determined using R123 as fluorescence dye. ATP levels determined using a bioluminescence assay. Data represent means ± SEM. *N* = 11 (six females, five males); one-way ANOVA with Tukey’s multiple comparison post test (*p < 0.05 against wild-type_(control)_; ^+^*p* < 0.05, ^++^*p* < 0.01 against Thy-1 AβPP_SL (control)_). CI complex I (NADH reductase, CII complex II (succinate dehydrogenase), CIV complex IV (cytochrome-c oxidase), ETS electron transfer system, AβPP beta-amyloid precursor protein

The complexes of the mitochondrial respiration chain (complex I, NADH reductase (CI); complex II, succinate dehydrogenase (CII); complex III, cytochrome-c reductase (CIII); complex IV, cytochrome-c oxidase (CIV)) build up a proton gradient at the inner mitochondrial membrane. The resulting membrane potential (MMP) finally represents the driving force for complex V of the mitochondrial respiration chain (F_1_/F_0_-ATPase (CV)) that produces ATP. Respiration of CIV of the mitochondrial respiration chain was significantly decreased in mitochondria isolated from brains of Thy-1 AβPP_SL_ mice (Fig. 6a). Consequently, MMP and ATP levels were significantly decreased in DBC isolated from Thy-1 AβPP_SL_ mice (Fig. 6b, c). All three parameters were reversed after MH84 treatment (Fig. 6a–c).

## Discussion

In this study, the effects of MH84 on mitochondrial dysfunction were investigated in 3-month-old transgenic Thy-1 AβPP_SL_ mice representing a model of early AD [37]. Since these mice develop typical Aβ plaques at the age of 6 months [39], we focused on soluble Aβ levels and confirm moderately elevated cerebral content of Aβ at an age of 3 months [39]. Moreover, levels of fl-AβPP and C99 were enhanced in brains of these mice as well. There is evidence that accumulation of Aβ, fl-AβPP, and C99, which are able to enter mitochondria and interact with mitochondrial proteins, might result in impaired mitochondrial function and energy metabolism [27, 48–52]. However, mitochondrial dysfunction might also be a result of lower levels of PGC-1α [32, 53]. There is convincing evidence for a link between PGC-1α and AD [32, 47], and numerous studies demonstrated that the pharmacological targeting of particular nuclear receptors is beneficial in mouse models of AD (reviewed in [13]). Decreased levels of PGC-1α have been reported in postmortem brain tissue of AD patients [33] and in a AβPPswe/PS1de9 mouse model of AD [54]. PGC-1α protein and mRNA levels were also found to be significantly decreased in the present study. PGC-1α has been characterized as one of the main regulators of PPARγ and as an essential initiator for mitochondrial biogenesis and respiration [55, 56]. Thus, decreased PGC-1α levels might play a role for impaired mitochondrial biogenesis [57] and mitochondrial dysfunction [37]. Accordingly, we determined the activity of the mitochondrial marker enzyme CS, which has been reported as one of the best markers of mitochondrial mass [58]. In our study, CS activity was significantly reduced in mitochondria isolated from brains of Thy-1 AβPP_SL_ mice, indicating reduced mitochondrial mass. This finding is in agreement with reduced PGC-1α levels in brain tissue samples isolated from these mice. However, Hauptmann et al. [37] detected no differences in the mitochondrial content in brains of Thy-1 AβPP_SL_ mice using MitoTracker Green fluorescence and PCR. On the other hand, reduced complex IV activity, MMP, and ATP levels are in accordance with this earlier publication [37] and other reports on mouse models of AD [59–61].

Mice were treated for 21 days with 12 mg/kg body weight (b.w.) MH84 using oral gavage. Pharmacokinetic properties of MH84 were investigated previously and we reported a maximum brain concentration of 320.64 ng/g [35]. MH84 is a small molecule that has been characterized as a dual PPARγ agonist and γ-secretase modulator in vitro [12, 34, 62]. However, our in-vivo data indicated modulation of β-secretase rather than γ-secretase: Treatment with MH84 significantly decreased the β-secretase cleavage product C99 and levels of soluble human Aβ40 in brain homogenates of Thy-1 AβPP_SL_ mice.

The present study also shows that MH84 reversed mitochondrial dysfunction and PGC-1α levels, decreased β-secretase processing of AβPP, and reduced levels of soluble Aβ40 in brains of transgenic Thy-1 AβPP_SL_ mice. These findings are in line with data for a cellular model of AD that we have been reported recently [36]. PGC-1α regulates a large set of mitochondrial genes as an essential initiator for mitochondrial biogenesis and respiration [55, 56]. Thus, increased CS activity and OXPHOS protein levels indicate that MH84 enhanced biogenesis of mitochondria by a PGC-1α-dependent mechanism and thereby compensates for mitochondrial dysfunction in brains of Thy-1 AβPP_SL_ mice. Enhanced MitoTracker Green (MTG) fluorescence after incubation of HEK-AβPPsw cells with MH84 further supports this hypothesis. MTG accumulates in mitochondria independently of the mitochondrial membrane potential and thus represents another suitable marker for the determination of mitochondrial content [63]. Katsouri et al. [19, 64] showed that PGC-1α reduces Aβ generation through a PPARγ-dependent mechanism and demonstrated recently that PGC-1α transfer reduces neuronal loss and Aβ generation by reducing β-secretase in an AD mouse model. Thus, PGC-1α might also be responsible for the β-secretase-related reduction of Aβ40 levels after treatment with MH84 in brains of Thy1-AβPP_SL_. However, in our study MH84 did not reverse enhanced BACE1 mRNA levels in brains of Thy1-AβPP_SL_ mice. On the other site, PGC-1α overexpression was reported to exacerbate Aβ and tau deposition in another transgenic mouse model of AD [65].

BDNF represents one of the most widely distributed and extensively studied neurotrophins in the mammalian brain. Its functions include developmental processes, regulation of synaptogenesis, and neuroprotection [66]. Decreased BDNF levels have been determined in brains of AD patients [67] and reduced expression of BDNF has been related to reduced PGC-1α levels in a mouse model of AD [47]. We report a significant downregulation of BDNF levels in brains of in brains of Thy-1 AβPP_SL_ mice, which was reversed after treatment with MH84. Thus, effects of MH84 might at least partly be related to enhanced BDNF expression although synaptic marker proteins were not changed.

## Conclusion

MH84 represents a dual γ-secretase/PPARγ modulator that improves mitochondrial dysfunction by enhancing mitochondrial dysfunction via a PGC-1α-dependent mechanism. Thus, MH84 seems to be a new promising therapeutic agent with approved in-vivo activity for the treatment of AD that could help to modify the disease progression in early stages.

## Additional files

Additional file 1: Figure S1. Representative NMR spectra of MH84 used for the current study. For analytical details please refer to [12]. (PDF 429 kb)

Additional file 2: Figure S2. Brain levels of the β-site of APP cleaving enzyme (BACE-1) mRNA. Expression levels of mRNA were normalized to PGK1 and B2M mRNA expression. Animals belonged to three different study groups (wild-type_(control)_, Thy-1 AβPP_SL (control)_, and treatment group Thy-1 AβPP_SL (MH84)_). Data represent means ± SEM. *N* = 11 (six females, five males); one-way ANOVA with Tukey’s multiple comparison post test (**p < 0.01, against wild-type_(control)_). (TIFF 562 kb)

Additional file 3: Figure S3. Western blot analysis of A brain-derived neurotrophic factor (BDNF), B Growth associated protein 43 (GAP43), and C synaptophysin in brain homogenate isolated from wild-type mice _(control)_, Thy-1 AβPP_SL (control)_, and MH-84-treated Thy-1 AβPP_SL (MH84)_ mice. Tubulin (A, C) and GAPDH (B) were used as loading controls. Data represent means ± SEM. *N* = 11 (six females, five males); one-way ANOVA with Tukey’s multiple comparison post test (***p < 0.001, **p < 0.01, against wild-type_(control)_; ^+^*p* < 0.05 against Thy-1 AβPP_SL (control)_). (TIFF 12802 kb)
