# Supplementary material for: MH84 improves mitochondrial dysfunction in a mouse model of early Alzheimer’s disease
Source: Alzheimers Res Ther. 2018 Feb 13;10:18. doi: 10.1186/s13195-018-0342-6 (PMC5809956; doi:10.1186/s13195-018-0342-6)
Supplement: Supplementary file 1 — Representative NMR spectra of MH84 used for the current study. For analytical details please refer to [12]. (PDF 429 kb) [file 13195_2018_342_MOESM1_ESM.pdf]

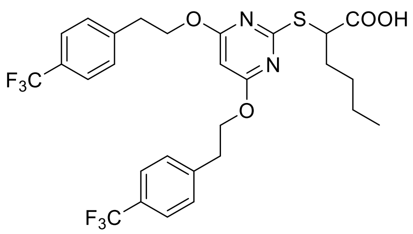

Chemical Formula: $C_{28}H_{28}F_6N_2O_4S$
Exact Mass: 602,17
Molecular Weight: 602,59

MS (ESI-):

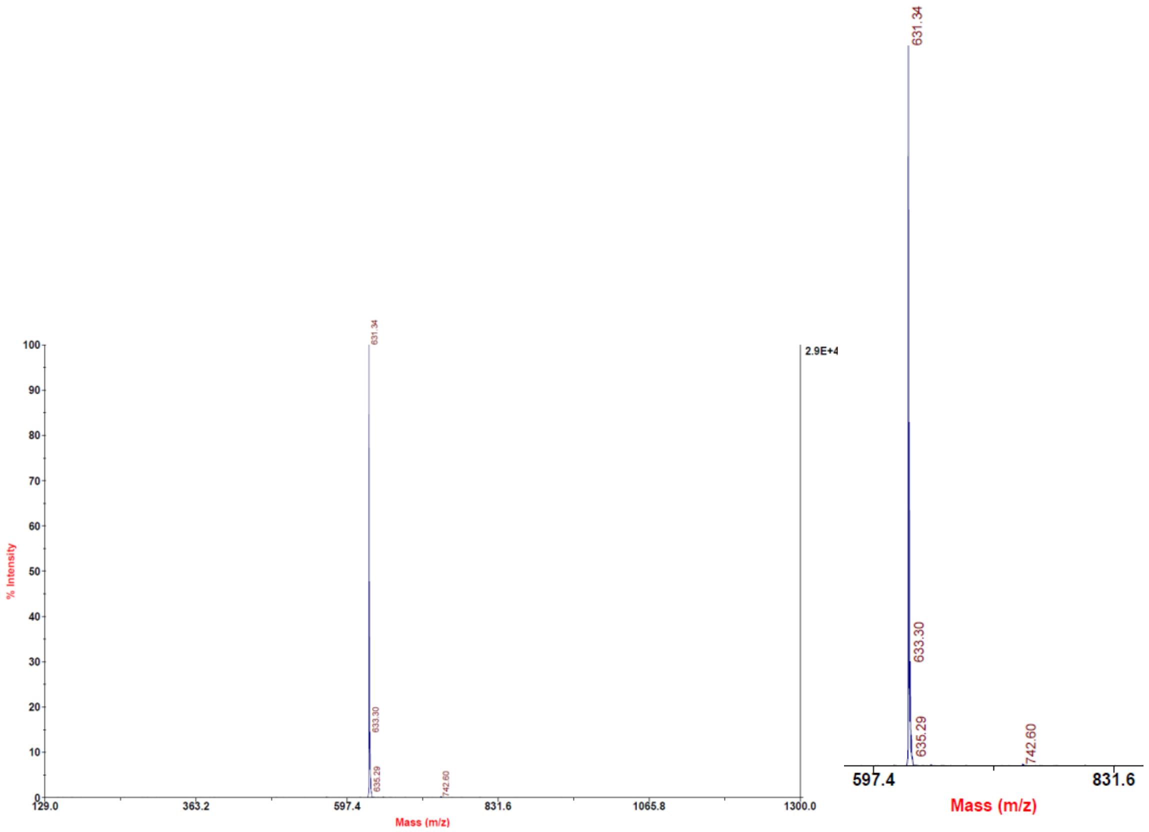

NMR:

^{1}H NMR (500 MHz, *CDCl$_3$*) δ = 7.49 (d, J = 8.1 Hz, 4H), 7.30 (d, J = 8.0 Hz, 4H), 5.61 (s, 1H), 4.51 – 4.38 (m, 4H), 4.27 (t, J = 7.4 Hz, 1H), 4.14 – 4.05 (m, 1H), 3.99 (dq, J = 10.8, 7.1 Hz, 1H), 3.02 (t, J = 6.8 Hz, 4H), 1.10 (dd, J = 9.4, 4.9 Hz, 4H), 0.89 – 0.79 (m, 8H).

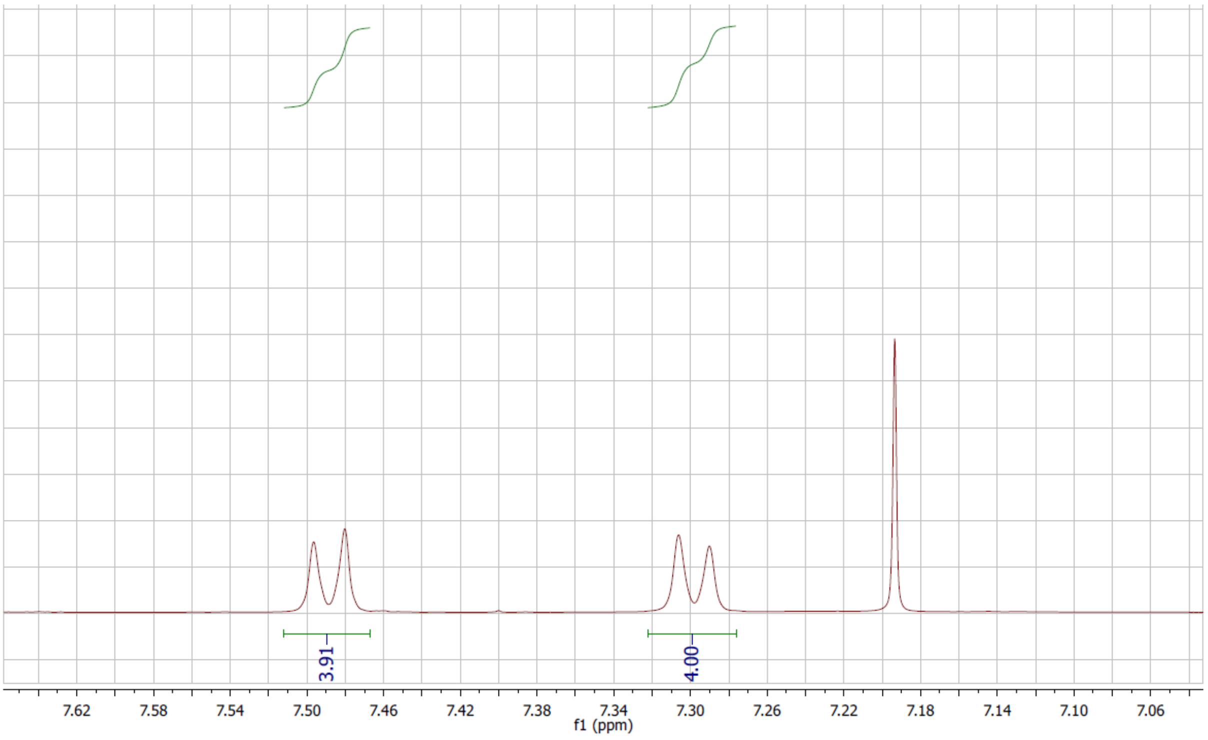
3.91
4.00
7.62
7.58
7.54
7.50
7.46
7.42
7.38
7.34
7.30
7.26
7.22
7.18
7.14
7.10
7.06
f1 (ppm)

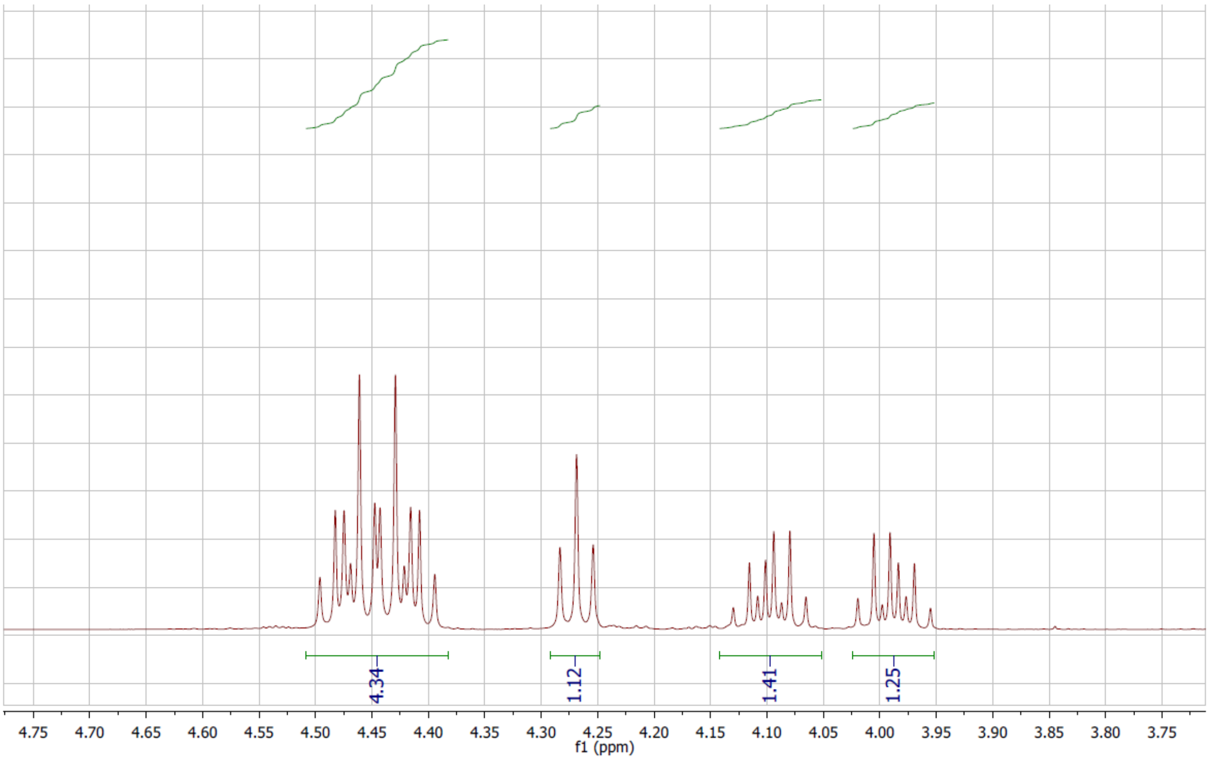
4.34
1.12
1.41
1.25
4.75
4.70
4.65
4.60
4.55
4.50
4.45
4.40
4.35
4.30
4.25
4.20
4.15
4.10
4.05
4.00
3.95
3.90
3.85
3.80
3.75
f1 (ppm)

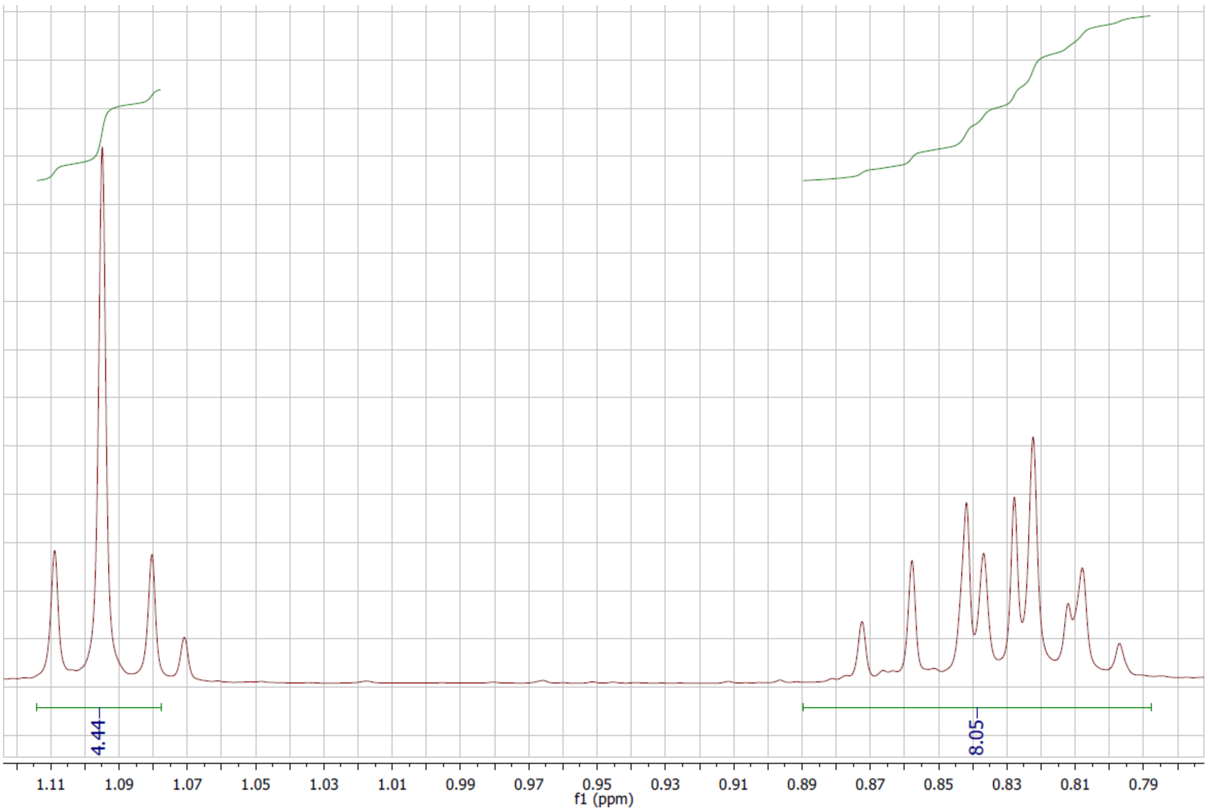
4.44
8.05
1.11 1.09 1.07 1.05 1.03 1.01 0.99 0.97 0.95 0.93 0.91 0.89 0.87 0.85 0.83 0.81 0.79
f1 (ppm)
