# Supplementary figures and images for: MH84 improves mitochondrial dysfunction in a mouse model of early Alzheimer’s disease
Source: Alzheimers Res Ther. 2018 Feb 13;10:18. doi: 10.1186/s13195-018-0342-6 (PMC5809956; doi:10.1186/s13195-018-0342-6)

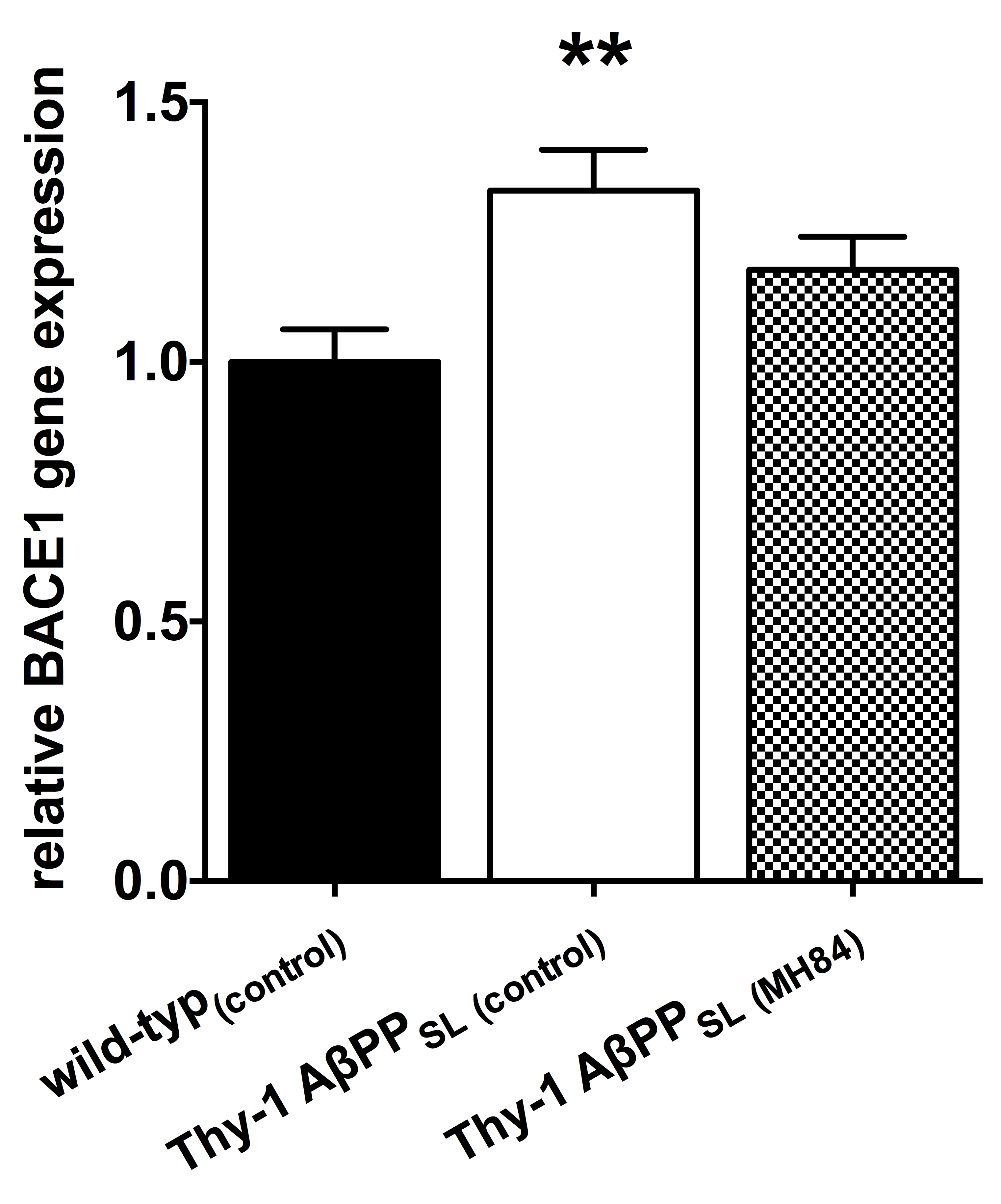

Supplement: Supplementary file 2 — Brain levels of the β-site of APP cleaving enzyme (BACE-1) mRNA. Expression levels of mRNA were normalized to PGK1 and B2M mRNA expression. Animals belonged to three different study groups (wild-type(control), Thy-1 AβPPSL (control), and treatment group Thy-1 AβPPSL (MH84)). Data represent means ± SEM. N = 11 (six females, five males); one-way ANOVA with Tukey’s multiple comparison post test (**p < 0.01, against wild-type(control)). (TIFF 562 kb) [file 13195_2018_342_MOESM2_ESM.tiff]

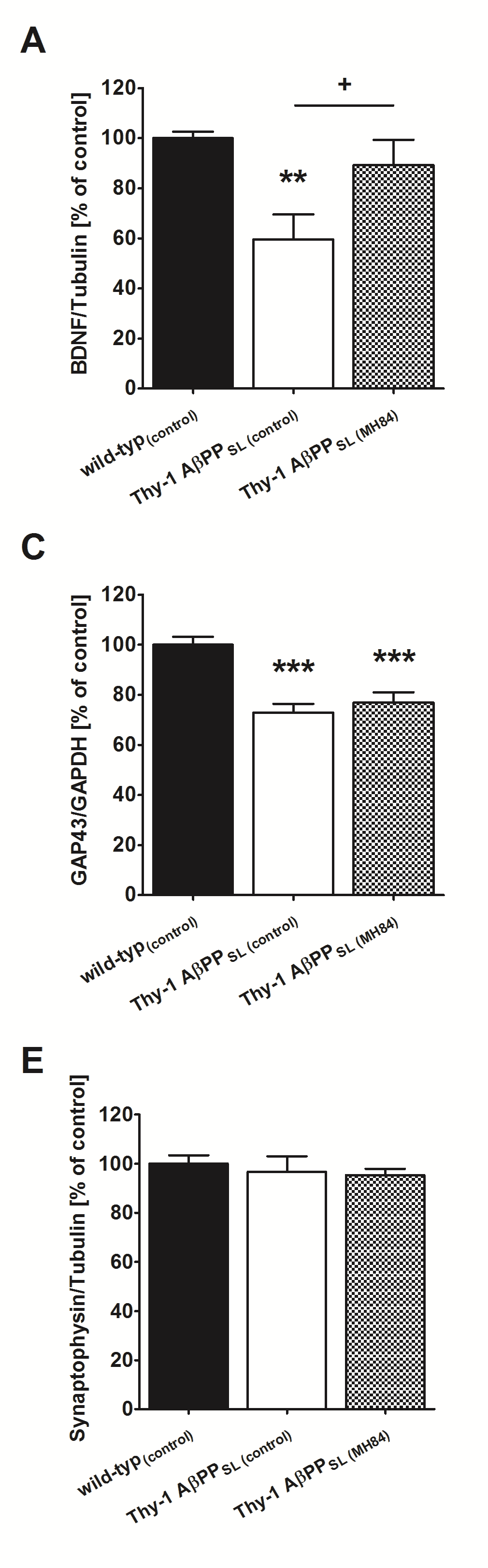

Supplement: Supplementary file 3 — Western blot analysis of A brain-derived neurotrophic factor (BDNF), B Growth associated protein 43 (GAP43), and C synaptophysin in brain homogenate isolated from wild-type mice (control), Thy-1 AβPPSL (control), and MH-84-treated Thy-1 AβPPSL (MH84) mice. Tubulin (A, C) and GAPDH (B) were used as loading controls. Data represent means ± SEM. N = 11 (six females, five males); one-way ANOVA with Tukey’s multiple comparison post test (***p < 0.001, **p < 0.01, against wild-type(control); +p < 0.05 against Thy-1 AβPPSL (control)). (TIFF 12802 kb) [file 13195_2018_342_MOESM3_ESM.tiff]
